# Exploring the Integration of Anthocyanins with Functional Materials in Smart Food Packaging: From Stabilization to Application

**DOI:** 10.3390/foods14162896

**Published:** 2025-08-20

**Authors:** Xiaowei Huang, Ke Zhang, Zhihua Li, Junjun Zhang, Xiaodong Zhai, Ning Zhang, Liuzi Du, Zhou Qin

**Affiliations:** 1 School of Food and Biological Engineering, Jiangsu University, 301 Xuefu Road, Zhenjiang 212013, China; 2 College of Food Science and Engineering, Nanjing University of Finance and Economics/Collaborative Innovation Center for Modern Grain Circulation and Safety, 128 North Railway Street, Nanjing 210023, China

**Keywords:** anthocyanins, polymer materials, nanomaterials, food packaging

## Abstract

Anthocyanins, the most ubiquitous water-soluble phytopigments in terrestrial flora, have garnered substantial attention in sustainable food packaging research owing to their exceptional chromatic properties, pH-responsive characteristics, and putative health-promoting effects. Nevertheless, their inherent chemical lability manifests as rapid chromatic fading, structural degradation, and compromised bioactivity/bioavailability, ultimately restricting industrial implementation and incurring significant economic penalties. Recent advances in stabilization technologies through molecular encapsulation within polymeric matrices or nanoscale encapsulation systems have demonstrated remarkable potential for preserving anthocyanin integrity while augmenting multifunctionality. The integration of anthocyanins into advanced functional materials has emerged as a promising strategy for enhancing food safety and extending shelf life through smart packaging solutions. Despite their exceptional chromatic and bioactive properties, anthocyanins face challenges such as chemical instability under environmental stressors, limiting their industrial application. Recent advancements in stabilization technologies, including molecular encapsulation within polymeric matrices and nanoscale systems, have demonstrated significant potential in preserving anthocyanin integrity while enhancing multifunctionality. This review systematically explores the integration of anthocyanins with natural polymers, nanomaterials, and hybrid architectures, focusing on their roles as smart optical sensors, bioactive regulators, and functional components in active and smart packaging systems. Furthermore, the molecular interactions and interfacial phenomena governing anthocyanin stabilization are elucidated. The review also addresses current technological constraints and proposes future directions for scalable, sustainable, and optimized implementations in food preservation.

## 1. Introduction

Food packaging, in its traditional sense, serves to shield food from external factors like dust, temperature, illumination, and humidity. This protection is vital for safeguarding the quality and safety of food products. Since the 20th century, food packaging technology has experienced unprecedented advancements. As consumer demand for enhanced food packaging has grown, active and smart packaging technologies have emerged. Active packaging systems are intended to control or slow the release of functional agents such as antimicrobials, dehumidifiers, and deaerators [1,2]. Alternatively, smart packaging systems provide consumers with information regarding environmental changes occurring within the food or its packaging through indicators [3], sensors [4], barcodes [5], and other means [6]. These systems offer opportunities to trace environmental variations and monitor the freshness of products throughout various stages, including production, inspection, regulation, and consumption. As a novel technology within the food industry, smart packaging equips consumers with intuitive and comprehensive information pertaining to the quality and freshness of packaged foods—thereby enhancing consumer trust. Colorimetric indicators, as a representative tool of smart packaging, are frequently employed to evaluate the freshness of meat products, fruits, vegetables, and dairy items. These indicators monitor color changes through cameras or other imaging devices under specific conditions, thereby facilitating the visual detection of spoilage [7]. The presence of organic acids [8], amines [9], carbon dioxide (CO_2_) [10], and sulfides [11] released due to microbial activity signifies spoilage. Advanced smart sensors can detect these changes in pH levels—whether acidic or alkaline—to signal degradation in food quality effectively.

The attractive color of anthocyanins derived from plant sources presents significant potential in colorimetric indicators of intelligent packaging systems. Furthermore, utilizing plant waste, such as fruit peels, as a precursor for extracting anthocyanins not only mitigates environmental pollution but also enhances economic benefits [12]. Importantly, compared to synthetic indicators, anthocyanins offer advantages including ease of preparation, non-toxicity, good biocompatibility, and healthcare functions [13]. However, the structural diversity regarding the type, location, and quantity of substituents on anthocyanin molecules renders them susceptible to unstable degradation and fading when subjected to external factors like enzymes, oxygen levels, temperature fluctuations, light, and pH variations [14]. Nevertheless, due to their diverse coloration at varying pH levels along with excellent biocompatibility and antibacterial properties, anthocyanins hold considerable promise within the realm of food packaging. Incorporating these compounds into packaging materials can facilitate monitoring changes in food quality during storage periods, thus providing qualitative or semi-quantitative information about the product’s condition. During storage intervals, anthocyanins interact with metabolites produced through food deterioration, allowing for visual assessment via colorimetric changes that indicate freshness levels in seafood, meat, dairy products, and fruits, among others [15]. In particular, researchers have been intrigued by the influence of pH on anthocyanins. An increase in pH can lead to hydration processes followed by tautomerization and further isomerization—accompanied by a series of predictable changes, including color loss [16].

Over the past few years, many researchers have concentrated on enhancing the stability of anthocyanins within food packaging systems through various strategies such as co-pigmentation [17,18], structural modification [19,20], and microencapsulation [6,21]. These efforts aim to develop active and smart packaging suitable for diverse types of food. The stability of anthocyanins is influenced by both intermolecular and intramolecular complexation [22]. Earlier studies indicate that functional materials—including natural polymers and nanoparticles—can adsorb or embed anthocyanins by forming hydrogen bonds, hydrophobic interactions, electrostatic interactions, and van der Waals forces [23,24]. This interaction effectively protects anthocyanins from external environmental interference. To date, polysaccharides [25], proteins [23], liposomes [26], and other natural polymers [27], along with nanomaterials [28] have been extensively utilized in the modification, encapsulation, and processing of anthocyanins to enhance their functionality. However, to our knowledge, a comprehensive review on the most recent advancements in functional materials aimed at encapsulating and stabilizing anthocyanins has yet to be conducted. In the context of food packaging, functional materials refer to substances or composites that possess specific, tailored properties to perform particular functions beyond mere structural support. These materials are designed to actively interact with the packaged contents or the surrounding environment to enhance the shelf life, quality, and safety of food products. Functional materials in food packaging can include, but are not limited to, bioactive polymers, nanomaterials, and hybrid systems that exhibit properties such as antimicrobial activity, moisture control, oxygen barrier properties, and even stimuli-responsive behaviors. These materials may also be engineered to incorporate natural bioactive compounds, like antioxidants or colorants, for smart and intelligent packaging applications. The integration of such materials aims to not only protect the food but also contribute to environmental sustainability, optimize preservation techniques, and ensure consumer safety. Consequently, this paper will concentrate on innovations that improve the stability of anthocyanins within active/smart packaging systems while also discussing recent advances in advanced functional materials and their interaction mechanisms. It will emphasize methods for enhancing both the stability of anthocyanins as well as the accuracy and sensitivity of detection techniques while aiming to reduce false positive rates. Additionally, potential challenges associated with these functional materials will be explored alongside prospective application scenarios—particularly concerning hazards and toxicity issues. In conclusion, integrating innovative functional materials with effective processing strategies can stabilize anthocyanins against environmental impacts, thereby augmenting their potential applications as value-added pigments and additives within the food industry.

## 2. The Properties of Anthocyanins in Food Packaging

### 2.1. Antioxidant Activities

**Figure 1:**
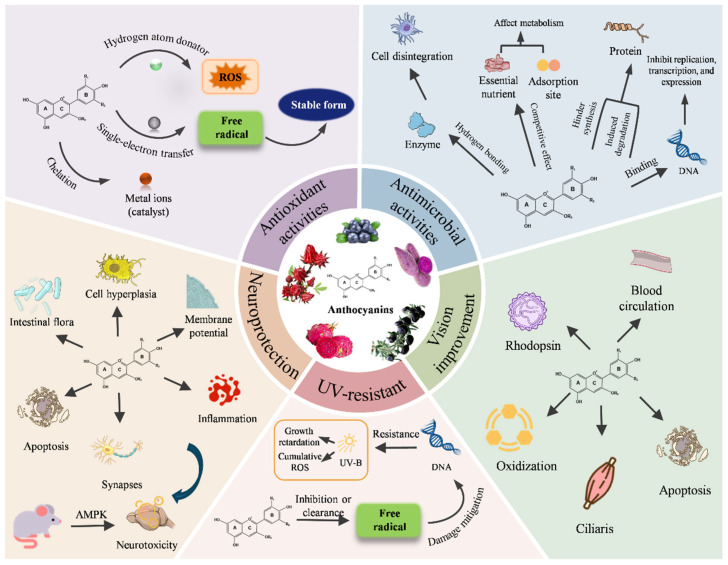
Schematic illustration of the multifunctional roles of anthocyanins (Some of the assets were sourced from Bioart).

The antioxidant properties of anthocyanins, which are flavonoid polyphenols, are of considerable significance in the context of food packaging [29,30]. These compounds exert their antioxidative effects primarily through hydrogen donation and single electron transfer mechanisms, both of which are crucial for neutralizing harmful radicals and oxidative intermediates [31]. The presence of ortho-phenol hydroxyl groups in anthocyanins enhances their radical scavenging capacity, enabling them to bind and neutralize free radicals, thus effectively mitigating oxidative stress [32]. Additionally, their ability to chelate metal ions that catalyze lipid oxidation further disrupts the chain reaction of lipid peroxidation, which is a key process in the deterioration of food quality [33]. To systematically elucidate the pleiotropic effects of anthocyanins, Figure 1 summarizes the key biological activities and their underlying mechanism. In food packaging, the integration of anthocyanins can be leveraged to enhance the shelf life and stability of packaged food products. By incorporating anthocyanins, food packaging materials can actively counteract oxidative degradation, thereby preserving the nutritional and sensory qualities of food. The antioxidant capacity of anthocyanins is directly correlated with their phenolic content, and as such, anthocyanin-enriched packaging materials can provide sustained protective effects against oxidation [34]. This property is particularly beneficial in the preservation of meat products, where lipid oxidation is a significant concern. Moreover, anthocyanins’ ability to modulate redox homeostasis and regulate inflammatory pathways adds a layer of functionality to food packaging, extending beyond mere oxidative protection. The potential for anthocyanins to influence metabolic and physiological processes further highlights their relevance in the development of smart food packaging systems. Such systems could incorporate anthocyanins to monitor and respond to environmental changes within the packaging, offering dynamic protection tailored to the specific needs of the packaged food. In summary, the antioxidative mechanisms of anthocyanins not only contribute to their role in promoting health and mitigating metabolic disorders but also enhance the functionality of food packaging by providing a natural, bioactive means of prolonging food quality and safety. The integration of anthocyanins into food packaging materials represents a promising approach for developing sustainable, efficient, and intelligent packaging solutions.

### 2.2. Antimicrobial Activities

The potential application of anthocyanins as antimicrobial agents in food packaging has garnered significant attention due to their bioactive properties and effectiveness against a wide range of pathogens. As systematically evaluated by [35], anthocyanins exhibit substantial antimicrobial and antiviral activity, suggesting their applicability in enhancing food safety and extending shelf life. The antimicrobial mechanisms of anthocyanins are diverse and depend on their structural characteristics, which may facilitate interactions with microbial enzymes and proteins, ultimately leading to the disruption of cellular integrity [36,37,38]. Anthocyanins, characterized by multiple phenolic hydroxyl groups within their flavonoid structure, can form hydrogen bonds with microbial enzymes and protein complexes. This interaction disrupts protein function, compromising the structural integrity of microbial cell walls, membranes, and cytoplasm, ultimately leading to bacterial disintegration [39]. This property makes anthocyanins an effective natural antimicrobial agent capable of inhibiting the growth of foodborne pathogens and thus plays a pivotal role in food preservation when incorporated into food packaging materials. In addition to direct bactericidal effects, anthocyanins also influence microbial metabolism. By altering membrane permeability and competing for essential nutrients, anthocyanins can inhibit microbial growth and proliferation [40]. The antimicrobial efficacy of anthocyanins has been further demonstrated through studies on specific sources such as Aronia melanocarpa. Ref. [41] found that anthocyanins from Aronia melanocarpa (AMAs) exhibited substantial inhibitory activity against pathogenic *Escherichia coli* by interfering with protein synthesis and DNA replication, leading to cellular apoptosis. Such mechanisms of action highlight the potential of anthocyanins to act as effective antimicrobial agents, providing an alternative to conventional synthetic antimicrobials. In conclusion, the incorporation of anthocyanins into food packaging represents a sustainable and effective approach to combating foodborne pathogens. Their broad-spectrum antimicrobial properties, coupled with their role in supporting probiotic activity, make anthocyanins a valuable asset in the development of eco-friendly, functional food packaging solutions that contribute to food safety and quality. Further research into the practical application of anthocyanin-based packaging could pave the way for novel packaging systems that align with growing consumer demand for natural, health-conscious products.

### 2.3. UV-Resistant

The ultraviolet-B (UV-B) radiation (280–320 nm) is a significant environmental stressor for plants, known to impede their growth and development, and in extreme cases, threaten their survival [42]. Prolonged UV-B exposure can also induce damage to the ocular system through the accumulation of reactive oxygen species (ROS) [43]. In this context, anthocyanins-flavonoid compounds that are responsible for the blue, purple, and red pigmentation in many plants—emerge as crucial molecules in mitigating oxidative damage induced by UV-B radiation. These compounds can either inhibit the formation of ROS or neutralize already-formed radicals, providing cellular protection against UV-induced oxidative stress [44]. Given that UV-B radiation can deteriorate the quality of food products through the degradation of nutrients and the formation of harmful substances, incorporating anthocyanins, particularly polyacylated forms, into food packaging materials could provide a natural barrier against UV-B radiation. Additionally, the incorporation of anthocyanins could offer a sustainable and eco-friendly alternative to synthetic UV-blocking agents, aligning with the growing demand for natural and biodegradable packaging solutions. Therefore, the UV-B shielding properties of anthocyanins present a promising approach to enhance food preservation and quality through innovative packaging technologies.

### 2.4. Neuroprotection

Research has demonstrated that anthocyanins exert beneficial effects on age-related cognitive decline, neurodegeneration, and memory impairment, potentially offering therapeutic avenues for conditions such as Alzheimer’s disease [45,46,47,48]. The mechanisms through which anthocyanins mediate these effects are multifaceted. Ref. [49] reviewed the interactions between anthocyanins and the gut–brain axis, highlighting their role in regulating intestinal microbiota and its metabolic byproducts, including the modulation of tryptophan catabolism. This process improves neurotransmitter activity and strengthens blood–brain barrier integrity, suggesting a novel therapeutic approach for neurodegenerative diseases. Further, extensive studies, including a meta-analysis by [50], have corroborated anthocyanins’ protective potential against Alzheimer’s-related pathologies. These include oxidative stress, astrocytic activation, cholinergic dysfunction, and neuroinflammation. Additionally, Ref. [51] demonstrated that anthocyanins could mitigate glutamate-induced neurotoxicity in vivo through an AMPK-dependent mechanism. In vitro studies suggest that their primary mode of action involves alleviating oxidative stress, reducing inflammation, and preventing neuronal degeneration [46]. Clinical animal trials further support the neuroprotective role of anthocyanins, showing delayed neurodegeneration and enhanced cognitive function when anthocyanin-rich products are consumed [52]. The neuroprotective properties of anthocyanins make them an invaluable addition to functional food packaging, which could help preserve the bioactive compounds and enhance their health benefits. Given the growing interest in food-based neuroprotective strategies, further investigation into the exact mechanistic pathways of anthocyanins and their incorporation into food packaging materials is crucial for advancing their practical applications in the prevention and management of cognitive impairments.

### 2.5. Vision Improvement

Studies have highlighted the ability of anthocyanins to alleviate retinal injury, mitigate visual fatigue, and enhance antioxidant defenses within the retina. For instance, blueberry extract, rich in phenolic compounds, has been shown to provide significant protection to retinal tissues by enhancing antioxidant and anti-apoptotic effects, thereby combating visual fatigue [53]. Similarly, Lycium berry extract has been demonstrated to reduce oxidative stress and protect photoreceptor cells from light-induced damage [54]. Further research suggests that anthocyanins may have a broader role in treating various visual disorders. For example, a study by [55] reported significant improvements in myopia and ocular moisture in participants consuming anthocyanin-rich preparations. In glaucoma patients, the intake of blackcurrant anthocyanins was shown to arrest visual field loss and improve ocular circulation [56]. Additionally, anthocyanins have been found to stimulate rhodopsin regeneration and enhance blood circulation within the eye, providing further evidence of their potential in treating conditions like myopia and glaucoma [57]. In conclusion, anthocyanins hold significant promise as nutraceutical agents for ocular health and food packaging applications. Their multifunctionality as both therapeutic agents for visual health and bioactive components in packaging systems underscores their potential impact in improving public health and food safety.

## 3. Functional Materials in Anthocyanin-Based Food Packaging

### 3.1. Natural Polymer Materials

#### 3.1.1. Polysaccharides

Polysaccharides refer to complex structures formed by the dehydration and condensation of more than twenty monosaccharide units [58]. They are widely distributed in nature, primarily found in plant vascular systems and reproductive structures, as well as in the mucus and shells of animals, and within and outside bacterial cells [59]. Consequently, polysaccharides are primarily classified into two distinct groups: animal-derived polysaccharides [60] and plant-derived polysaccharides [61]. Animal-derived polysaccharides include chitosan, chondroitin sulfate, and keratan sulfate; whereas plant-derived polysaccharides encompass starch, pectin, and oligo-fructose. Different types of polysaccharides exhibit physical or chemical interactions with anthocyanins that lead to the formation of distinct microstructures within systems, thereby altering the stability and functionality of anthocyanins. Chitosan, a positively charged polysaccharide, is derived either through chitin deacetylation or fungal extraction processes [62]. Its significant antibacterial properties have been confirmed through various studies [63,64]. The antimicrobial action mechanism is primarily mediated through electrostatic attraction between the protonated amino groups of chitosan and anionic components of microbial cell membranes, leading to subsequent membrane disruption and cellular lysis [65]. Ref. [66] engineered a novel biopolymer composite film by incorporating MXene and tannic acid into a chitosan matrix, demonstrating enhanced antimicrobial and antioxidative capabilities that effectively prolong the preservation of bananas and grapes. In acidic conditions, chitosan undergoes protonation, leading to increased solubility, which in turn affects the functionality of colorimetric films in low pH environments [67]. Therefore, chitosan-based indicator films can effectively monitor the accumulation of acids during the spoilage processes of foods, including fresh-cut fruits, milk, and vegetables. Ref. [68] developed and characterized a pH-sensitive smart film made from sodium alginate/quaternary ammonium salt-chitosan composites loaded with anthocyanins for monitoring milk freshness. The investigation revealed that anthocyanin encapsulation within the sodium alginate/quaternary ammonium salt-chitosan complex is mediated by electrostatic forces and hydrogen bond formation. Over a 72 h period of monitoring milk freshness, it was observed that as the freshness decreased, the composite film color shifted from purple to red, indicating good stability and effectiveness in assessing milk freshness. The anthocyanin indicators can function through interactions mediated by chitosan and endogenous compounds in meat matrices, thereby conveying freshness information to consumers. Ref. [69] developed an innovative chitosan-based smart packaging film responsive to volatile amines, incorporating butterfly pea flower extract as a pH-sensitive indicator. Their findings demonstrated that anthocyanin-chitosan interactions are predominantly mediated through hydrogen bonding within the polymer matrix. The developed film demonstrated dual functionality, serving as both a visual freshness indicator through distinct color transitions across four beef quality stages (fresh → sub-fresh → initial spoilage → fully spoiled) and an effective antimicrobial agent. The described innovations demonstrate successful integration of chitosan with functional additives to create multifunctional platforms combining preservation and smart sensing. Future developments should prioritize enhancing environmental adaptability with pH-stable matrix design, creating multi-stimuli responsive systems for diverse food matrices, and adopting sustainable manufacturing processes. These advancements, integrating food chemistry, microbiology, and smart packaging engineering, must balance technological complexity with cost-effectiveness for commercial success.

Gum is an edible natural substance exuded from the roots or stems of trees and shrubs; it forms shell-like crystals upon drying [70]. Owing to its remarkable antioxidative and antimicrobial characteristics, this material has found widespread application in food packaging for spoilage prevention and shelf-life extension. Ref. [71] developed a fully natural food packaging film utilizing materials such as Astragalus gum, chitosan nanoparticles, a wild grape extract (Sardasht black) rich in anthocyanins, and aluminum oxide nanoparticles. This biodegradable film demonstrates strong antioxidant and antimicrobial properties, displaying a noticeable color transition displaying a visible naked-eye chromatic transition from red to blue over a wide pH spectrum (2–12) in various buffer solutions. The pioneering work by [72] demonstrated the novel application of Persian gum, either in isolation or combined with maltodextrin, as an effective encapsulation matrix for anthocyanins, presenting a viable strategy for developing stable coloring agents.

As abundant biopolymers, sodium alginate and carboxymethyl cellulose represent eco-friendly polysaccharides with inherent biocompatibility [73,74]. Ref. [75] investigated the application of anionic polysaccharides—such as maltodextrin, gum arabic, xanthan gum, and carboxymethyl cellulose—as coating agents for microencapsulating anthocyanins extracted from Aronia melanocarpa fruit. Throughout storage, encapsulated anthocyanins demonstrated markedly higher stability (88–91%) compared to their free counterparts, thereby demonstrating the efficacy of these polysaccharides as encapsulating agents. However, polysaccharide-based packaging materials often exhibit mechanical weaknesses and susceptibility to collapse. To mitigate this issue, incorporating natural or recycled fibers can enhance mechanical properties while preserving desirable attributes such as biodegradability and renewability. Zhang and colleagues developed a novel intelligent indicator aerogel utilizing black goji anthocyanins, sodium alginate, carboxymethyl cellulose, and natural fibers through a freeze-drying method. This aerogel was specifically applied to monitor the freshness of fish [76].

Polysaccharides hold significant promise for enhancing food packaging, not only through their role in encapsulating anthocyanins but also by serving as functional ingredients in films with antimicrobial, antioxidant, and other protective properties. By utilizing polysaccharides in food packaging, we are not only addressing the need for effective spoilage prevention but also contributing to the reduction in plastic waste by providing biodegradable, eco-friendly alternatives to synthetic packaging materials. This innovative approach aligns with growing environmental concerns and paves the way for the development of sustainable, multifunctional food packaging solutions.

#### 3.1.2. Proteins

Proteins are biological macromolecules that consist of hundreds of amino acids, exhibiting unique structures and a wide range of functional properties [77]. Proteins fall into three principal classifications: animal proteins (e.g., whey protein, serum albumin, ferritin, casein), plant proteins (e.g., soy protein, rice protein), and others. Due to the moderate polarity of anthocyanins and their natural affinity for proline-rich proteins, these compounds can spontaneously bind with protein carriers within food matrices. Encapsulating anthocyanins within proteins can enhance their stability and bioactivity, including attributes such as color retention, thermal stability, and antioxidant capacity [78]. Therefore, utilizing the interaction between proteins and pigments to enhance the physicochemical stability of pigments represents a direct and effective strategy. The biomolecular interactions between anthocyanins and edible proteins—whether derived from plants or animals—are primarily non-covalent in nature [79]. Given the distinctive properties of anthocyanin molecules alongside variations in side-chain groups and amide bonds present in different proteins, these intermolecular forces include hydrogen bonding, hydrophobic effects, ionic interactions, and van der Waals attractions [79]. Stable anthocyanin-protein complexes may arise from covalent bonding via electron sharing between anthocyanins and nucleophilic amino acid residues [80]. It should be emphasized that in covalently bound complexes, the coloration associated with anthocyanins may diminish due to oxidation reactions leading to quinone formation [81].

Ref. [82] systematically examined the non-covalent binding interactions occurring between rose-derived anthocyanin extracts and isolated whey protein across different pH environments. At both pH 3.0 and pH 7.0, the systems demonstrated non-covalent interactions characterized by two distinct binding sites that involved hydrogen bonds and van der Waals forces. To enhance grape skin anthocyanin utilization in cereal products, Ref. [83] investigated their gliadin interactions and grape skin anthocyanin extracts (GSAE). The results from molecular docking revealed that Gli interacts with various anthocyanin monomers at different binding sites, primarily relying on hydrogen bonds as well as hydrophobic interactions. These findings provide additional evidence for the formation of Gli-GSAE complexes while demonstrating the viability of anthocyanins as naturally derived coloring agents. Stable anthocyanin-protein aggregates enhance anthocyanin stability while boosting protein functionality, notably in foaming, solubility, and emulsification performance. This strategy has been demonstrated to be effective for color stabilization while providing additional advantages concerning the functional properties of plant bioactive components, such as antioxidant activities, and food structure. To further enhance the application of anthocyanins in the food industry, Ref. [84] modified black rice anthocyanins using caffeic acid esterase to produce acylated anthocyanins (Ca-An). Combining experimental and computational analyses, they examined Ca-An/SPI binding mechanisms. Structural characterization showed Ca-An complexation altered protein conformation, increasing secondary structure mobility, particularly at 7S and 11S regions, where enhanced hydrophilicity was observed. Molecular docking and molecular dynamics simulations indicated that SPI and Ca-An primarily interact through hydrogen bonds and van der Waals forces to form a stable complex. The 11S subunit accommodates Ca-An within its hydrophobic cavity, forming stable hydrogen bonds, whereas 7S interacts spontaneously through van der Waals interactions. These findings illustrate the stable binding of SPI-Ca-An and provide valuable insights into how anthocyanins interact with other proteins, thereby facilitating further investigations into the mechanisms underlying modified anthocyanins’ interactions with biomolecules. Ref. [85] investigated covalent bonding between β-lactoglobulin (β-Lg) and anthocyanins derived from purple sweet potato peel, as well as their implications for developing a green/smart salt-responsive biosensor aimed at monitoring fish freshness. Docking simulations combined with multispectral analyses revealed that β-Lg successfully underwent phenolic coupling with anthocyanins, while also interacting with pullulan (Pul) through hydrogen bonding and other intermolecular forces. This interaction significantly enhanced the antioxidant properties, antimicrobial activity, moisture resistance, and thermal stability of the β-Lg/Pul biosensor.

#### 3.1.3. Liposomes

Liposomes are minute vesicles composed of phospholipids and cholesterol, characterized by a bilayer phospholipid structure [86]. Due to their biocompatibility, biodegradability, low toxicity, and capacity to encapsulate both hydrophilic and lipophilic drugs, liposomes have found extensive applications across the pharmaceutical, food, and cosmetic industries. Furthermore, liposomal encapsulation systems have been thoroughly investigated within the food and agriculture sectors for developing delivery systems that stabilize unstable compounds such as antimicrobial agents, antioxidants, flavorings, and bioactive elements while preserving their functionality [87,88]. Ref. [89] developed blueberry anthocyanin microcapsules (BAM) utilizing blueberry anthocyanin as the core material combined with sodium alginate as the wall material through a coacervation-emulsification method. Blueberry anthocyanin liposomes (BAL) were prepared by dispersing soy lecithin and cholesterol via the thin-film dispersion technique. Optimization through univariate analysis and response surface methodology revealed the optimal preparation conditions, yielding encapsulation efficiencies of 96.14% for BAM and 81.26% for BAL. The findings indicate that both BAM and BAL effectively maintain the stability of blueberry anthocyanins without significant differences in stability indicators. It provides a theoretical foundation for developing systems aimed at enhancing the stability of anthocyanins, thereby improving their bioavailability upon human ingestion. Ref. [90] prepared grape skin anthocyanin nano-liposomes using a thin-film ultrasonic dispersion method; this encapsulation system demonstrated improved stability of anthocyanins under various temperature and light conditions. Their study offers valuable theoretical and practical insights into improving the stability and bioavailability of anthocyanins, supporting their use in health supplements and functional foods. Current literature shows that the development of nanoemulsion/nanoliposome systems for stabilizing anthocyanin glycosides is still in its early stages. Future investigations should prioritize the optimization of encapsulating materials and preparation techniques to create liposomal systems characterized by high encapsulation efficiency, improved stability, and reduced particle sizes, which could facilitate their potential clinical applications moving forward.

#### 3.1.4. Natural Polymer Composites

**Figure 2:**
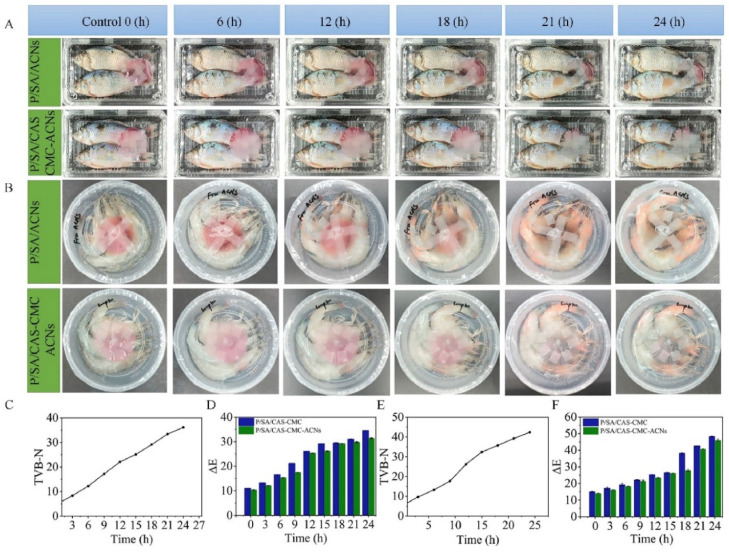
(**A**) Colorimetric films for monitoring fish freshness. (**B**) Colorimetric film for monitoring shrimp freshness. (**C**) TVB-N value change in fish. (**D**) ΔE value changes in films for fish freshness monitoring. (**E**) TVB-N value change in shrimp. (**F**) ΔE value changes in films for shrimp freshness monitoring. Adapted with permission from Ref. [92]. 2025 Elsevier.

Anionic polysaccharides can interact electrostatically with positively charged proteins, thereby enhancing the stability of dispersion systems through the formation of ionic bonds [91]. This interaction subsequently promotes the bioavailability and activity of bioactive compounds. The utilization of protein-polysaccharide nanocomposites for the encapsulation of anthocyanins has been extensively documented, offering a novel approach to active and smart food packaging. Arif Rashid and colleagues employed pullulan and sodium alginate as raw materials, combining them with casein carboxymethyl cellulose (CMC) nanocomposites loaded with anthocyanins to develop a novel smart colorimetric film. As shown in Figure 2, the application of composite films for freshness monitoring of fish and shrimp resulted in a notable color change from pink to dark gray. This finding was ascribed to the presence of carboxyl groups in CMC, which can interact with anthocyanins via ionic bonds formed with metal cations, thus facilitating their encapsulation. Additionally, hydrogen bonding between components within the composite film further enhanced its structural integrity while improving barrier properties, mechanical strength, and thermal stability [92]. Aqueous two-phase systems (ATPS) are based on phase separation phenomena observed in aqueous solutions, where two immiscible phases—typically water and polymer solution—are formed [93]. ATPS-based microcapsules have garnered considerable attention due to their favorable biocompatible microenvironment; however, high concentrations of synthetic polyethylene glycol and dextran used in conventional ATPS hinder their application within the food industry. Jiang and colleagues developed an innovative food-grade ATPS microcapsule by leveraging electrostatic interactions along with hydrogen bonding between chitosan and a mixture comprising collagen plus pectin. Pectin was key in stabilizing anthocyanins, leading to an encapsulation efficiency of 92.58% and a drug-loading capacity of 12.34 g/100 g. The developed microcapsules demonstrated the most robust morphology, highest stability, and smoothest interface at pH 6. This phenomenon results from the electrostatic attraction between metal-cationic anthocyanins and anionic pectin [94]. This innovative ATPS microcapsule presents a promising alternative to conventional systems for encapsulating hydrophilic bioactive compounds. However, the degradation of bioactive components within the microcapsules in vivo remains an area that requires further investigation.

Owing to the degradation, aggregation, fusion, oxidation, and hydrolysis of phospholipids, the half-life of liposomes in the gastrointestinal tract is typically limited [95]. Recent studies highlight the potential of polysaccharide polymer coatings to improve the stability and therapeutic efficacy of liposomes. Ref. [96] developed chitosan-coated nanoliposomes using an advanced heating method to encapsulate caffeine and anthocyanins from Hibiscus flowers for beverage fortification. Under optimized conditions (lecithin-to-cholesterol ratio of 1:3 and wall-to-core ratio of 2.16), the liposomes achieved caffeine encapsulation efficiency of 66.73% and anthocyanin glycoside encapsulation efficiency of 97.03%. Similarly, Ref. [97] investigated chitosan-coated canola lecithin nanoliposomes for encapsulating anthocyanins with the aim of enhancing their stability. Rheological analysis indicated that the incorporation of chitosan altered the behavior of the nanoliposomes from that characteristic of Newtonian fluids to shear-thinning behavior. Their findings suggest that chitosan-based systems are effective in encapsulating anthocyanins while improving their stability, thereby rendering them promising candidates for applications in both pharmaceutical and food industries.

Previous studies have shown that chitosan effectively protects liposomes by reducing oxygen exposure and aggregation, thereby enhancing their stability in the digestive tract. Chitosan-coated liposomes are increasingly used in the food industry to encapsulate bioactive compounds like anthocyanins, curcumin, green tea extracts, carotenoids, eugenol, and α-linolenic acid. In order to protect anthocyanins under acidic conditions and facilitate a higher loading of the anthocyanin precursor within proteins, Ref. [98] proposed a “flavonoid front-end stability strategy”. The anthocyanin nanoliposomes (Lip@ACN) were synthesized under acidic conditions to ensure the amphiphilic properties of the anthocyanin prototype. Casein methacrylate hydrogel (GelCSMA) was developed by modifying casein, which is otherwise unsuitable for hydrogel formation, in order to prevent the detrimental effects of alkaline conditions on anthocyanin stability. Subsequently, Lip@ACN was incorporated into the hydrogel matrix to form the Lip@ACN/CSMA composite. Furthermore, the photo and thermal storage stability improved by 36.50% and 30.71%, respectively. The front-end stability anthocyanin loading system effectively preserves the amphiphilicity of the anthocyanin precursor while mitigating the adverse effects associated with alkaline conditions. This innovative approach offers a novel and efficient method for stabilizing anthocyanins.

The integration of anthocyanins with natural polymeric matrices, including polysaccharides, proteins, and lipids, has demonstrated remarkable potential in developing multifunctional packaging systems. These bio-based composites not only provide real-time freshness indication through pH-responsive colorimetric changes but also enhance food preservation through their inherent antioxidant and antimicrobial properties. Polysaccharides, proteins, liposomes, and natural polymer composites offer versatile, sustainable solutions for food packaging. These materials help encapsulate anthocyanins, enhancing their stability and functionality while contributing to the reduction in plastic waste through biodegradable alternatives. Their use in food packaging aligns with the growing need for eco-friendly and multifunctional solutions in the food industry.

### 3.2. Engineering Nanomaterials

#### 3.2.1. Nanoemulsions

Nanoemulsions are colloidal dispersions composed of oil and water phases, with droplet sizes ranging from 10 to 100 nm [99]. The physicochemical properties of nanoemulsions, including droplet size distribution and interfacial layer characteristics (such as thickness, composition, and electrical properties), significantly influence the encapsulation efficiency and controlled release of anthocyanins. Integrating anthocyanin-loaded nanoemulsions into food-grade emulsion systems can enhance their controlled release and stability, thereby improving the antimicrobial and antioxidant properties of active compounds [100]. Ref. [101] developed a photochromic film for food applications (specifically shrimp) using pectin-based nanoemulsions that encapsulated anthocyanins extracted from Hibiscus fruit peel. Spectroscopic analysis revealed significant hydrogen bonding interactions as well as compatibility among the film components. Notably, the smart film demonstrated visible color changes from cherry/pink to yellow/brown, effectively serving as an indicator for monitoring shrimp spoilage. Ref. [102] prepared microemulsions and nanoemulsions encapsulating Carissa spinarum plant extract to investigate the degradation kinetics of phenolic and flavonoid compounds. The results indicated that nanoemulsions exhibited higher quorum-sensing (QS) inhibition activity against *Vibrio harveyi* compared to microemulsions. This study represents the first report utilizing more stable microemulsions and nanoemulsions as alternatives to traditional antibiotics in QS inhibition research. However, one limitation associated with nanoemulsions is their relatively short shelf life due to uncontrolled or unfavorable conditions under which encapsulated compounds may be released.

#### 3.2.2. Nanoparticles

##### Metallic Nanoparticles

**Figure 3:**
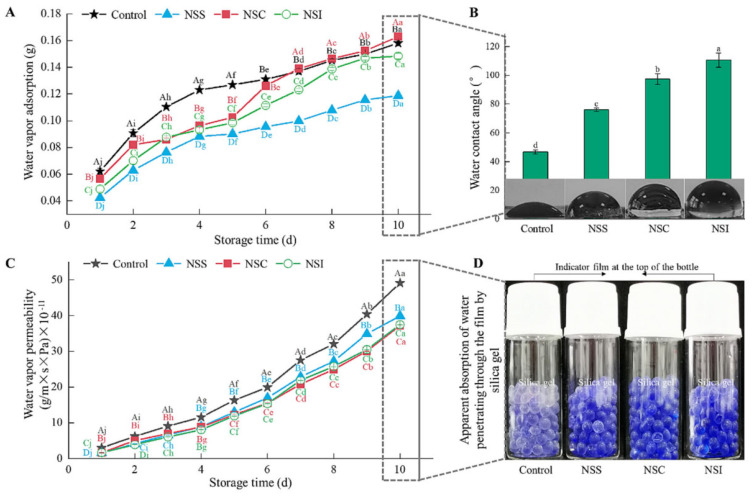
Water barrier properties of indicator films stored at 4 °C for 10 days at 100% humidity. (**A**) Water vapor adsorption; (**B**) Contact angle; (**C**) Water vapor permeability; (**D**) Water absorption through the film as indicated by silica gel. Adapted with permission from Ref. [111]. Letters mean NSS, NSC, and NSI refering to the alginate/anthocyanin/cellulose nanocrystal/nanosilica indicator films prepared using the spraying (S), coating (C), or impregnation (I) procedures, respectively. 2025 Elsevier.

**Figure 4:**
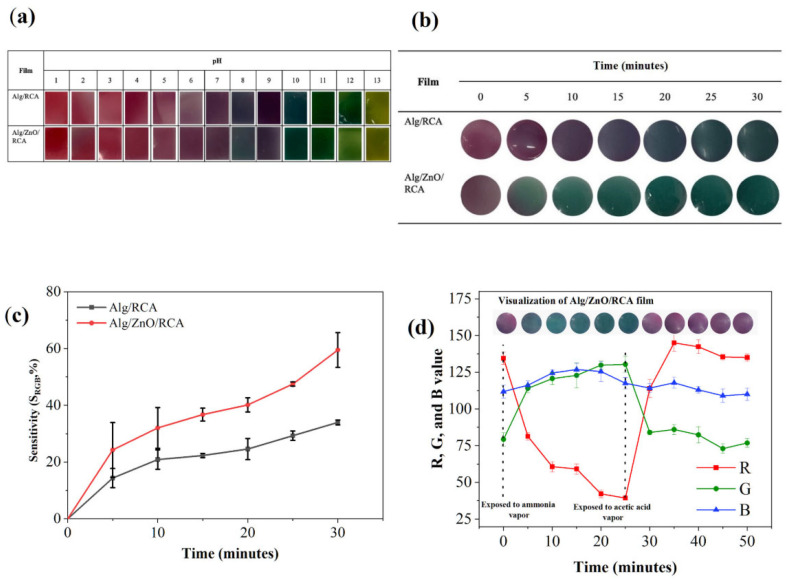
(**a**) pH-dependent colorimetric transition across the pH range 1–13, (**b**) Comparative ammonia vapor (15 mM, 30 min) sensitivity between Alg/RCA and Alg/ZnO/RCA composites, (**c**) Quantitative colorimetric analysis, and (**d**) Visual representation of Alg/ZnO/RCA films exposed to 15 mM ammonia and 25% acetic acid vapors with associated RGB color coordinates [113].

Metal-based nanoparticles consist of metal cores functionalized with surface-bound molecules [103]. These nanoparticles, including those of silver (Ag), zinc oxide (ZnO), titanium dioxide (TiO_2_), and silicon dioxide (SiO_2_), are capable of stabilizing and supporting water-soluble compounds such as anthocyanins. These nanoparticles exhibit distinctive properties such as magnetism, photocatalysis, ultraviolet (UV) shielding, fluorescence, and antimicrobial activity [104]. Metal-based nanosensors are frequently integrated with anthocyanins in the development of smart food packaging systems. These sensors are capable of detecting variations in temperature, humidity, light, and gas levels—indicators of food spoilage—and can trigger the release of antimicrobial agents when necessary [105]. Nano-TiO_2_ is particularly recognized for its effective absorption and scattering of UV light; this property enables it to safeguard anthocyanins and plant extracts by minimizing their exposure to UV radiation [106]. Ref. [107] developed a bilayer visual indicator film for monitoring Penaeus chinensis freshness, combining κ-carrageenan and butterfly pea anthocyanins with varying TiO_2_ nanoparticle concentrations and agar. The κ-carrageenan-anthocyanin layer acts as the indicator, while the TiO_2_-agar layer provides protection. This design minimizes anthocyanin leaching in aqueous solutions and improves the film’s photostability. Ref. [108] studied the impact of TiO_2_ nanoparticle size on chitosan/corn zein films with carrot anthocyanins. They found that TiO_2_ improved thermal stability, light stability, hydrophobicity, and UV barrier properties but reduced mechanical strength and swelling. Notably, their study revealed that smaller TiO_2_ nanoparticles exhibited stronger interactions with the film components, leading to a more compact network structure and reduced aggregate sizes. This research confirmed that the size of TiO_2_ nanoparticles is closely associated with the performance and stability of anthocyanin-based composite films. Nano-SiO_2_ is a widely utilized nanomaterial known for its biodegradability, biocompatibility, and cost-effectiveness [109]. It comprises Si-O-Si and Si-CH_3_ groups, which exhibit low affinity for water binding, while also providing a rough microstructure at the micron or nanometer scale [110]. Ref. [111] developed hydrophobic films by spraying, coating, and impregnating nano-SiO_2_ into a film containing anthocyanins as an indicator. Figure 3 demonstrates that nano-SiO_2_ addition substantially improves film performance, exhibiting enhanced mechanical strength, superior water vapor barrier properties, and markedly increased surface hydrophobicity. ZnO nanoparticles are widely employed in anthocyanin-integrated smart packaging applications owing to their exceptional biocompatibility, non-toxicity, antimicrobial activity, antioxidant capacity, and remarkable photoelectric characteristics with high electron transfer efficiency [112]. Ref. [113] engineered a pH-responsive alginate-based film through the integration of red cabbage anthocyanin and ZnO nanoparticles. As demonstrated in Figure 4, the film exhibited distinct color transitions from purple (fresh) to blue-green (spoiled) upon shrimp storage testing, while maintaining non-cytotoxic properties toward RSC-96 cells and enhanced detection sensitivity through ZnO incorporation. Ref. [114] fabricated a novel pH-sensitive film utilizing chitosan, methylcellulose, ZnO nanoparticles, and black goji berry anthocyanin as the matrix. ZnO nanoparticle integration conferred UV-shielding properties to the film matrix and anthocyanin components via hydrogen bonding and multiple interfacial interactions, significantly improving the system’s photostability. Numerous studies have highlighted the potential of nanoparticles as enhancers for anthocyanin-based food freshness indicator packaging.

##### Porous Organic Frameworks (POFs)

Porous organic frameworks (POFs), which mainly encompass metal–organic frameworks (MOFs) and covalent organic frameworks (COFs), have emerged as effective platforms for gas storage and release due to their high surface area and tunable composition [115]. The incorporation of natural dyes into POFs can yield stable structures, allowing target gases to diffuse through both the surface and pores of the POFs. This results in an enrichment effect that enhances the colorimetric response of natural gas sensors. Ref. [116] developed a green nanoparticle-based colorimetric sensor to monitor seafood freshness. They modified six types of POFs with four food-safe natural dyes, resulting in 24 nanopigments. The color enhancement of these POFs was assessed before and after nanoparticle formation, identifying four nanopigments with high sensitivity. However, the results revealed that only certain modified nanopigments exhibited the desired color sensitivity, highlighting the challenges in selecting suitable POF materials for sensor applications.

**Figure 5:**
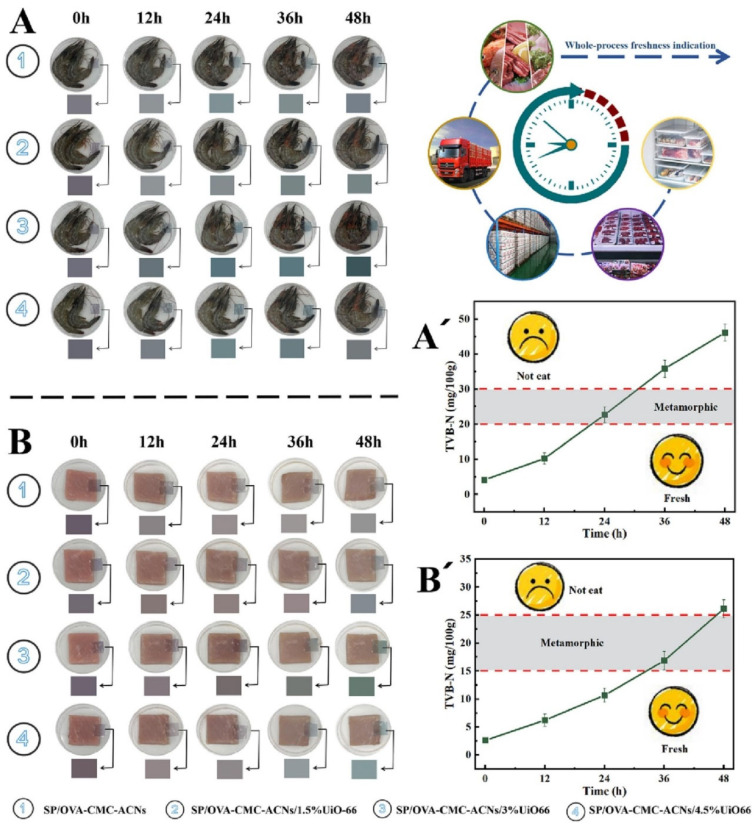
The temporal colorimetric responses of composite films to shrimp (**A**) and pork (**B**) spoilage, accompanied by corresponding TVB-N concentration profiles (**A’**,**B’**) throughout the storage period. Adapted with permission from Ref. [123]. 2025 Elsevier.

MOFs are porous crystalline materials composed of metal ions coordinated to organic ligands [117]. MOFs and their derivatives, as emerging nanomaterials, are attracting significant attention. Due to their stability, high porosity, excellent adsorption capacity, antibacterial activity, and catalytic properties, MOFs have gained significant attention as versatile porous matrices for incorporating diverse molecular species [118]. Ref. [119] enhanced the stability of anthocyanin encapsulation by ion-exchanging acetate ions into the pores of γ-cyclodextrin-MOF (CD-MOF), resulting in increased anthocyanin adsorption and a maximum loading of 83.7% within one minute. The CD-MOFs provided improved protection under heat and light exposure, doubling anthocyanin stability compared to free anthocyanins, making them promising materials for food packaging. The coordinatively unsaturated Cu (II) sites within the Cu-MOF demonstrate pronounced ammonia affinity, triggering distinct chromatic transitions that enable real-time ammonia monitoring [120]. Ref. [121] developed a smart active nanocomposite film by incorporating a Cu-MOF with antimicrobial and ammonia-responsive properties into starch and polyvinyl alcohol matrices. This film exhibited rapid color changes upon exposure to ammonia and successfully monitored shrimp freshness, extending shelf life at both 4 °C and 28 °C. The UiO-66 series, a zirconium-based MOF, is distinguished by its exceptional thermal and chemical stability, which allows it to endure harsh conditions such as high humidity, acidic and alkaline solutions, and various organic solvents. This characteristic renders it particularly suitable for the fabrication of composite films [122]. Ref. [123] fabricated a novel film system for real-time freshness monitoring of shrimp and pork products, utilizing a composite matrix of ovalbumin-carboxymethylcellulose embedded with anthocyanin and zirconium-based UiO-66 nanoparticles. The addition of 3% UiO-66 promoted uniform distribution and pore formation within the films, enhancing their gas adsorption capacity by tenfold and improving their response to low ammonia concentrations. As demonstrated in Figure 5, UiO-66 not only stabilized anthocyanins but also enhanced the gas adsorption properties of the films. This significantly improved the sensitivity of these freshness indicator films for detecting food spoilage. Notably, UiO-66-NH_2_ possesses multiple functional groups (such as -NH_2_ and -COO^−^) that confer excellent UV absorption and fluorescence properties. Ref. [124] developed an innovative pH colorimetric sensor based on UiO-66-NH_2_ for rapid detection of ammonia levels by integrating sodium alginate, red cabbage anthocyanin, and UiO-66-NH_2_. The sensor showcased significant UV absorption coupled with interaction with anthocyanins, resulting in enhanced stability while minimizing anthocyanin leakage. The developed sensor exhibited high selectivity for amine and ammonia vapors, demonstrating reliable anti-interference capability and enabling visual, early-stage spoilage detection in shrimp under different temperature conditions. The main challenge in MOFs’ industrial production lies in the high costs and limited availability of precursors. Therefore, developing cost-effective MOF precursors for efficient mass production is crucial for their widespread industrial application. Ongoing research suggests that MOF-based functional materials have the potential to outperform traditional alternatives, laying a solid foundation for advancements in rapid and highly sensitive food safety detection methods.

As the metal-free counterpart of MOFs, COFs are distinguished by their periodic dynamic covalent bonds within intramolecular structural units [125]. This unique architecture endows COFs with several advantages, including high surface area, tunable pore size, and chemical stability, rendering them highly appealing for a wide range of applications such as targeted drug delivery and controlled release [126,127], as well as enhancing the sensitivity and selectivity of electrochemical biosensors [128,129]. In addition, time and temperature are critical factors influencing the freshness of perishable foods. Ref. [130] has developed a novel COFs-based thermal history indicator (THI) that exhibits irreversible color changes, enabling real-time, accurate, nondestructive, and intuitive detection of cumulative variations in time and temperature during the distribution of products within the food supply chain. However, COF-based THIs remain in their early stages of development. Currently, there is a need for COFs with color-changing activation energies that align with the metamorphic activation energies of various foods; furthermore, the actual monitoring performance when applied to real food items has yet to be thoroughly investigated.

##### Quantum Dots

Quantum dots (QDs) are spherical, low-dimensional semiconductor nanocrystals with particle sizes typically less than 10 nm. They consist of a semiconductor core and surface functional groups [131]. In QDs, when all three spatial dimensions fall below a critical size, electron movement becomes confined within the boundaries of the nanocrystals, resulting in unique quantum confinement effects. These effects endow QDs with UV-blocking capabilities, fluorescence properties, high stability, antibacterial activity, and antioxidant characteristics [131,132]. Research has primarily concentrated on the controlled release and drug delivery [133,134]; detection of food analytes [135,136]; and active/smart packaging systems [137,138]. The diverse array of functional groups present on QDs—such as carboxyls, hydroxyls, carbonyls, and epoxy groups—significantly enhances their binding affinity and loading capacities [139].

**Figure 6:**
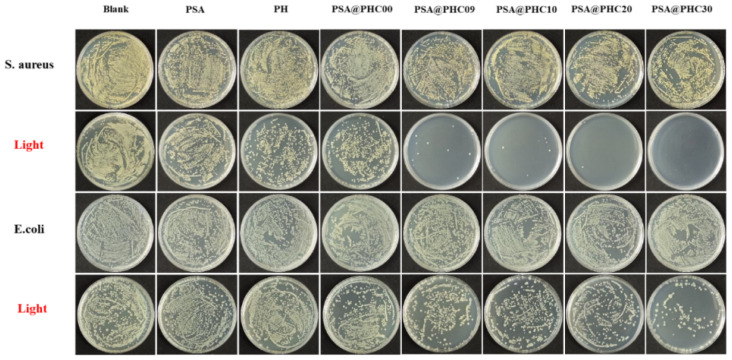
The antimicrobial performance of fabricated films against *E. coli* and *S. aureus* in both photonic and non-photonic environments, accompanied by fluorescence visualization of bacterial viability in PSA@PHC30 composite at λ_ex_ = 410 nm. Adapted with permission from Ref. [142]. 2025 Elsevier.

In recent years, carbon quantum dots (CDs) synthesized from biological waste have gained significant traction as active fillers in edible and degradable biopolymer membranes [140]. These CDs enhance the functionality of the membranes while simultaneously mitigating environmental pollution and improving economic efficiency. Ref. [141] synthesized CDs from purple hull pistachio by-products and incorporated them into chitosan-anthocyanin films. The films provided 100% UV protection, exhibited strong antibacterial activity (22.1 ± 0.24 mm against *Staphylococcus aureus* and 20.36 ± 0.21 mm against *E. coli*), and demonstrated significant antioxidant properties (82.3 ± 0.1% DPPH, 90.6 ± 0.1% ABTS). Additionally, visible discoloration served as a reliable freshness indicator, extending fish shelf life by 12 days. Ref. [142], on the other hand, prepared anthocyanins alongside CDs derived from barley bran, demonstrating that these CDs could generate ROS upon exposure to light—thereby inhibiting bacterial proliferation effectively. The incorporation of CDs into nanofiber-based smart double-layer films significantly prolonged food shelf life under illuminated conditions (Figure 6). Without proper stabilizers, CDs exhibit aggregation behavior, leading to compromised biofunctional performance in both antimicrobial and antioxidant activities. The incorporation of metal nanoparticles can stabilize and improve the functionality of CDs. Ref. [143] developed a pH-sensitive colorimetric bilayer film with chitosan-supported purple carotenin as the inner indicator and gellan-supported magnesium-doped CDs as the outer layer. Mg-CDs formed hydrogen bonds with the substrate, enhancing color stability, thermal stability, and significantly improving antioxidant and antibacterial properties. Researchers led by Ajahar Khan engineered an innovative carrageenan film integrated with anthocyanin-rich purple cabbage extracts and ZnO-incorporated carbon dots for smart packaging applications. The film exhibited UV-A and UV-B barrier efficiencies of 85.2% and 99.4%, scavenged ABTS and DPPH radicals (99% and 58.6%, respectively), and showed strong antibacterial activity, inhibiting L. monocytogenes and reducing *E. coli* by 8.1 log CFU/mL after 12 h. It holds significant potential for freshness monitoring, spoilage reduction, and shelf-life extension [144]. Natural pigments combined with CDs sourced from kitchen waste represent innovative tools for developing smart and sustainable packaging systems tailored for future applications within the food industry.

The integration of biomass-derived CDs into advanced biopolymer matrices offers a multifunctional platform for food packaging innovation, addressing critical needs in food preservation and safety through their dual roles as structural modifiers and bioactive components, while meeting growing consumer demands for sustainable packaging technologies. Despite advancements in the preparation, modification, and performance evaluation of QDs, their practical application within the food industry remains nascent. An urgent challenge is to develop more efficient and safe modification strategies aimed at enhancing the functional capabilities of QDs. Numerous studies underscore the potential of QDs as functional materials for active or smart packaging solutions, presenting a viable alternative to synthetic plastics in mitigating environmental accumulation issues along with associated health risks.

##### Biopolymeric Nanoparticles

Due to the increasing focus on environmental protection and public health, natural biopolymers and pigments have gained significant attention in the development of biodegradable and renewable active smart packaging films. Natural biopolymer nanomaterials represent an innovative class of green nanomaterials, distinguished by their non-toxicity, high biocompatibility, and large specific surface area [145]. Studies have revealed that biopolymer-based nanoencapsulation significantly improves anthocyanin stability while maintaining their pH-responsive characteristics, offering an efficient stabilization strategy for these bioactive compounds [146]. Ref. [147] developed a corn starch/chitosan composite membrane with rosin-coated potato amylopectin nanoparticles as a pH indicator to assess shrimp freshness. The color transition from pink to colorless to yellow effectively reflects freshness levels. Their study showed that amylopectin nanoparticle incorporation formed new hydrogen bonds with the substrate, enhancing the film’s mechanical properties, light stability, and water vapor barrier performance. Cellulose nanocrystals—rigid, rod-like structures derived from plant sources with a high Young’s modulus—are typically obtained through acid hydrolysis or chemical oxidation of cellulose macromolecules [148]. Ref. [149] developed a pH-responsive multifunctional packaging film using cellulose nanocrystals, which significantly enhanced its antioxidant, antibacterial, and color sensitivity properties. Furthermore, the film is fully biodegradable, meeting environmental sustainability standards. This highlights the potential of cellulose nanocrystals in developing eco-friendly and functional packaging materials.

#### 3.2.3. Nanoclays

Nanoclays exhibit nanoscale layered structures that provide numerous advantages over both natural and synthetic materials, primarily due to their abundance, environmental sustainability, and water solubility [150]. Their high adsorption capacity is attributed to the extensive surface area of both internal and external sites, which facilitates the stripping process. This unique property enhances the intercalation of organic molecules, including anions and cations, thereby promoting efficient adsorption of natural dyes. Researchers have developed biohybrid materials that leverage intercalation to adsorb anthocyanins onto nanoclays, effectively shielding bioactive compounds from interference by external environmental factors [151]. Among various nano-clay materials, montmorillonite (Mt) stands out due to its naturally occurring inorganic composition comprising silicon tetrahedrons and aluminum octahedrons arranged in a distinctive 2:1 layered structure. This configuration imparts a negative net charge to its surface, enabling the adsorption of exchangeable cations. Given these properties, Mt demonstrates significant potential for application in the development of bio-hybrid pigments [152]. Ref. [153] successfully stabilized anthocyanin extracts within Mt through electrostatic interactions, confirming that Mt exhibits favorable adsorption properties for anthocyanins and represents a novel type of biohybrid material. This method not only aids in the recovery of anthocyanins but also enhances the stability of their colorimetric properties. Montmorillonite has been shown to form polymerized anthocyanin nanosystems through two mechanisms: embedding bioactive compounds within the interlayer spacing of Mt and facilitating van der Waals force interactions between polymerized anthocyanin molecules and the outer surface of Mt [154]. Ref. [155] prepared color-changing biodegradable composite films by incorporating anthocyanins derived from wolfberry with Mt to create organic/inorganic hybrid pigments, which were subsequently blended into sodium alginate. The findings indicate that anthocyanins are immobilized on the Mt surface via electrostatic attraction forces and cation exchange, as well as being embedded within the interlayer spaces of Mt. Notably, the weather resistance of anthocyanin molecules was significantly enhanced across various environmental conditions. More importantly, in comparison to the control group, the composite film exhibited favorable mechanical properties and reversible acid-induced discoloration behavior.

Halloysite nanotubes (HNT) are 1:1 aluminosilicate clay materials characterized by a nanotube-like structure composed of five to six layers [156]. In addition to their tubular morphology, the unique physicochemical properties of HNT make them appealing as nanocarriers for drug delivery systems and diverse biomedical applications [157]. Furthermore, they serve effectively as carriers for organic pigments such as anthocyanins. Ref. [158] demonstrated that synthesizing inorganic/organic hybrid pigments loaded with anthocyanins on HNT is an effective strategy for enhancing the thermal stability, UV resistance, and acid stability of natural anthocyanin pigments. Sepiolite is a fibrous nano-clay composed of trioctahedral layered silicates featuring magnesium at its octahedral center. The low crystallinity and small particle size inherent in sepiolite confer excellent adsorption capabilities [159]. Ref. [160] has shown that sepiolite interacts with hibiscin through non-covalent bonds or van der Waals forces; notably, this interaction may primarily occur at a superficial level. Ref. [161] immobilized anthocyanins from purple cabbage within sepiolite nanoclays to create pH-sensitive membranes for monitoring milk spoilage. These films exhibited a visible spectrum band gap and stable, rapid color changes in response to pH variations, enabling real-time freshness monitoring without prolonged milk contact. While this approach holds promise for creating advanced food packaging nanosystems that can stabilize and encapsulate active compounds, concerns regarding their potential food toxicity may restrict their application as materials intended for direct food contact. Existing research reveals a significant knowledge gap regarding the toxicological assessment of nanoscale packaging composites in food applications, necessitating comprehensive investigations to establish their safety profile and practical viability as anthocyanin-based polymeric nanomaterials for food packaging.

#### 3.2.4. Polymer Nanomicelles (PNMs)

Polymer nanomicelles (PNMs) are nanomaterials derived from amphiphilic polymers, typically ranging in size from 1 to 200 nm [162]. These nanoparticles comprise two functional components: an “inner core” and an “outer shell”. The outer shell, composed of hydrophilic segments such as polyethylene glycol, plays a critical role in modulating pharmacokinetic properties in vivo. Conversely, the inner core, which consists of hydrophobic components, is essential for drug encapsulation, stabilization of the nanomicelle structure, and controlled release of therapeutics [163]. It has been reported that PNMs exhibiting high stability demonstrate favorable biocompatibility in cancer therapy applications [164]. Furthermore, PNMs possess the capability to encapsulate poorly soluble drugs, thereby preventing unintended interactions and biodegradation within environmental contexts while enhancing their biological efficacy [165]. The unique structural configuration of PNMs offers an ideal platform for anthocyanin stabilization, addressing their inherent limitations such as pH sensitivity and low bioavailability. Moreover, the synergistic combination of PNMs’ drug delivery potential with anthocyanins’ inherent bioactivities (antioxidant, antimicrobial, and anti-inflammatory properties) opens new avenues for developing multifunctional active packaging materials. Future research should focus on optimizing PNM-anthocyanin formulations for specific food applications, investigating their safety profiles, and exploring scalable production methods.

These nanomaterials, including nanoparticles and nanocomposites, improve the stability, sensitivity, and responsiveness of anthocyanins to environmental changes, making them ideal for use in food preservation systems. By integrating such materials, food packaging not only gains enhanced protective properties but also facilitates real-time monitoring of freshness, contributing to better quality control.

## 4. Conclusions and Prospects

Anthocyanins have demonstrated significant advantages in the food industry, particularly in food preservation and freshness monitoring. This is primarily attributed to their unique biological properties, which include color rendering, antibacterial and antioxidant effects, as well as potential health benefits such as UV resistance, neuroprotection, and enhanced visual function. This paper reviews innovative research on functional materials utilized in active/smart packaging systems. These materials encompass polysaccharides, proteins, liposomes, and nanomaterials such as nanoemulsions, nanoparticles, nanofibers, nanoclays, and nanomicelles. Notably, preparations that involve loading anthocyanins onto functional materials—especially metal-based nanomaterials—can effectively stabilize and protect anthocyanins while facilitating their controlled release. This process enhances the bioactivity and bioavailability of anthocyanins to varying extents. The interactions between these functional materials and anthocyanins are characterized by hydrogen bond formation, van der Waals forces, electrostatic interactions, and other proximity effects that contribute to the stabilization and protection of anthocyanins. This strategy has been successfully implemented in the production of packaging films for active/intelligent packaging systems. The results indicate that these films exhibit excellent response sensitivity alongside good color stability and remarkable antibacterial efficacy. These findings hold significant implications for addressing challenges related to anthocyanins within domains such as food safety and healthcare.

To effectively harness the advantages offered by functional materials in food packaging applications, several critical issues must be addressed. Foremost among these concerns is the safety of functional materials that may migrate from packaging systems into food products, along with their potential toxicological impacts. Further research is essential to elucidate the impact of ingesting these functional materials on consumers and to investigate the mechanisms and implications of bacterial resistance to nanoparticles. Concurrently, in order to mitigate environmental pollution and safety risks, it is crucial to thoroughly evaluate the biodegradation performance of certain functional materials. Future investigations should prioritize uncovering new characteristics of these functional materials as well as their compatibility with bioactive substances such as anthocyanins. Moreover, a deeper understanding of the interaction mechanisms between bioactive compounds and functional materials will emerge as a pivotal topic for future research. In terms of processing methods that incorporate active ingredients like anthocyanins, challenges persist regarding their sustainable development and economic viability for industrial applications. Therefore, interdisciplinary collaboration is imperative to identify opportunities for preserving the properties of active ingredients such as anthocyanins while simultaneously developing environmentally friendly, cost-effective, and scalable methods or technologies that facilitate the successful integration and widespread application of functional systems containing anthocyanins in food packaging and healthcare.
